# Characterization of the Proteins Involved in the DNA Repair Mechanism in *M. smegmatis*

**DOI:** 10.3390/ijms21155391

**Published:** 2020-07-29

**Authors:** Angela Di Somma, Carolina Canè, Antonio Moretta, Arianna Cirillo, Franz Cemič, Angela Duilio

**Affiliations:** 1 Department of Chemical Sciences, Università Federico II di, 80126 Naples, Italy; angela.disomma@unina.it (A.D.S.); cane@ceinge.unina.it (C.C.); 2 Istituto Nazionale Biostrutture Biostrumentazioni, INBB, 00136 Rome, Italy; 3 CEINGE Biotecnologie Avanzate, 80145 Naples, Italy; cirilloa@ceinge.unina.it; 4 Department of Science, Università degli Studi della Basilicata, 85100 Potenza, Italy; antonio.moretta@unibas.it; 5 Department of Mathematics, Natural Sciences and Computer Science, University of Applied Sciences Giessen, Wiesenstr. 14, 35390 Giessen, Germany; franz.cemic@mni.thm.de

**Keywords:** alkylating agents, DNA-damaging, adaptive response, *Mycobacterium smegmatis*, molecular docking, gel filtration chromatography

## Abstract

Several alkylating agents that either occur in the environment or are self-produced can cause DNA-damaging injuries in bacterial cells. Therefore, all microorganisms have developed repair systems that are able to counteract DNA alkylation damage. The adaptive response to alkylation stress in *Escherichia coli* consists of the Ada operon, which has been widely described; however, the homologous system in *Mycobacterium tuberculosis* (MTB) has been shown to have a different genetic organization but it is still largely unknown. In order to describe the defense system of MTB, we first investigated the proteins involved in the repair mechanism in the homologous non-pathogenic mycobacterium *M. smegmatis*. Ogt, Ada-AlkA and FadE8 proteins were recombinantly produced, purified and characterized. The biological role of Ogt was examined using proteomic experiments to identify its protein partners in vivo under stress conditions. Our results suggested the formation of a functional complex between Ogt and Ada-AlkA, which was confirmed both in silico by docking calculations and by gel filtration chromatography. We propose that this stable association allows the complex to fulfill the biological roles exerted by Ada in the homologous *E. coli* system. Finally, FadE8 was demonstrated to be structurally and functionally related to its *E. coli* homologous, AidB.

## 1. Introduction

The DNA molecule is a crucial target for several alkylating molecules that either occur in the environment or are self-produced. They can react with nucleophilic sites on DNA bases and cause covalent modifications, cytotoxic damage and impair cell survival [1]. Alkylating agents have electrophilic properties that make them able to directly interact with DNA or RNA, causing mutations during replication and transcription [2]. A number of sites prone to alkylation have been identified on DNA bases including the guanine oxygen site at position 6. Methylation of this oxygen generates O^6^-methylguanine (O^6^-MeG), which prevents the correct base pairing and leads to mutations during DNA replication. Other well-known alkylation products are N^7^-methylguanine (N^7^-MeG), N^3^-methyladenine (N^3^-MeA), N^1^-methyladenine (N^1^-MeA), N^3^-methylcytosine (N^3^-MeC), and O^4^-methyltimine (O^4^-MeT) [3,4].

Bacteria have developed repair systems that are able to counteract DNA alkylation damage and avoid cell death. The adaptive response to alkylation stress in *Escherichia coli* relies on the so-called Ada operon which has been widely described [5,6]. The protein products of this operon provide increased resistance when bacterial cells are exposed to sublethal doses of alkylating agents. The sensor enzyme of the defense mechanism is the Ada protein, a methyltransferase that is able to remove and transfer methyl groups from damaged DNA bases to a cysteine residue in its active site. Following methylation, Ada undergoes conformational changes to become a positive regulator for the expression of the *ada* gene itself and the other repair genes located within the operon, *alkA, alkB* and *aidB* and it is involved in the adaptive response [5,7,8].

AlkA is a DNA-glycosylase responsible for the removal of 3-methyl adenine and several other nitrosation products, such as N^7^-methyladenine, N^3^-methylguanine, N^7^-methylguanine, by hydrolyzing their N-glycosidic bonds. The dioxygenase AlkB catalyzes the hydroxylation of the methyl group of methylated DNA bases resulting in the subsequent formation of succinate and CO_2_, and bringing the nitrogen bases back to their original state [9,10]. Finally, the flavoprotein AidB belongs to the family of acyl-Coenzyme A (acyl-CoA) dehydrogenases, which are endowed with unspecific DNA binding properties and weak dehydrogenase activity. This protein has been shown to play a protective role by inactivating alkylating agents before they react with DNA [11,12].

**Figure 1:**
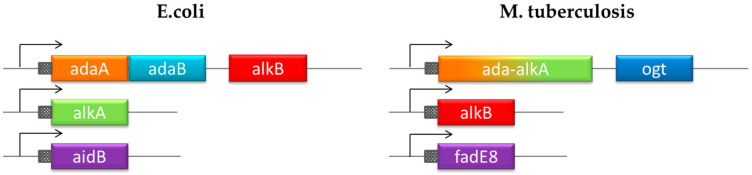
Chromosomal organization of the adaptive response to alkylation stress in *E. coli* and *Mycobacterium tuberculosis* (MTB).

Unlike *E. coli*, the DNA protection system in *Mycobacterium tuberculosis* (MTB) has not been investigated and it is still largely unknown. Recent studies led to the identification of four genes encoding proteins involved in an adaptive response mechanism homologous to the defense system described in *E. coli* but with different gene organization (Figure 1) [13,14]. Exposure of MTB to methylating molecules strongly increases the transcription of *ada-alkA* and *ogt* genes, which demonstrates an inducible response to methylating agents. Moreover, Ogt was identified as a methyltransferase that is homologous to the B domain of the Ada protein in *E. coli* that transfers the O^6^-alkyl group from modified guanines to a strictly conserved cysteine residue (Cys 126) in the protein active site [15,16]. On the basis of sequence homology, Ada-AlkA should consist of the A domain of the *E. coli* Ada protein fused to the AlkA sequence.

With the aim of describing the defense system in MTB, we first investigated the specific role of the proteins involved in the repair mechanism in the homologous non-pathogenic mycobacterium *M. smegmatis*. Most of these proteins were produced in *E. coli* and characterized in terms of structure and function. The biological function of Ogt was elucidated by a functional proteomic approach designed to identify its protein partners in vivo, which suggested a possible interaction with Ada-AlkA. The specific association of these proteins to form a stable complex was validated both in silico and in vitro by docking calculations and gel filtration chromatography using the recombinant forms of Ada-AlkA expressed in *E. coli* and fully characterized. Finally, FadE8, which showed sequence homology with the AidB protein from *E. coli,* was cloned, expressed in *E. coli* and characterized for its FAD binding, dehydrogenase activity and DNA binding capabilities. The investigation of the DNA repair system in *M. smegmatis* allowed us to characterize proteins that are also present in MTB and play key roles in survival and protection mechanisms. Since these proteins are absent in humans, they might represent excellent targets for new potential therapeutic approaches.

## 2. Results and Discussion

The comparing the DNA repair network in MTB with the homologous system in *E. coli*, highlights the uniqueness of the Ogt protein. On the basis of its primary structure, this protein is homologous to the B domain of the *E. coli* protein Ada, whereas the A domain is fused to the DNA-glycosylase AlkA (Figure 1).

Besides its methyltransferase activity, Ada is the sensor protein responsible for the transcription activation of the entire operon following alkylation stress. In a previous paper, Ogt was demonstrated to be a methyltransferase with DNA binding capabilities [15]. This unusual gene organization prompted us to investigate the respective roles of Ogt and Ada-AlkA and the other proteins involved in the repair mechanism in *M. smegmatis*, a non-pathogenic mycobacterium homologous to MTB.

### 2.1. Investigation on the Ogt Protein

**Figure 2:**
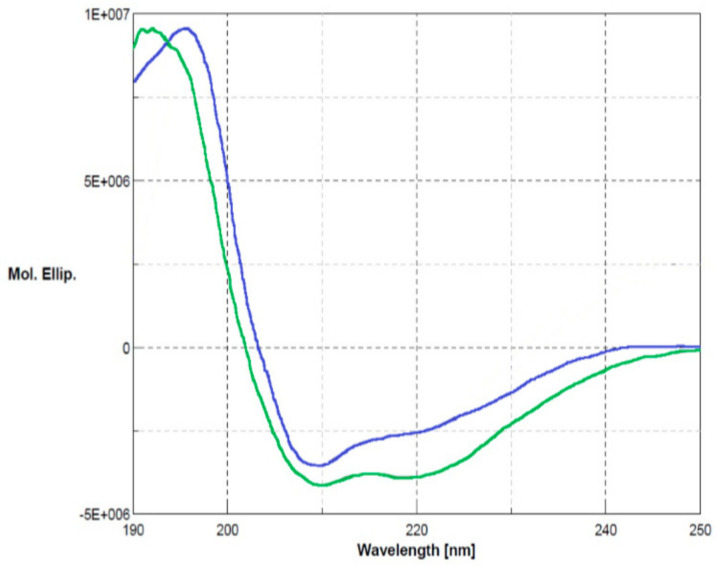
Circular dichroism analyses of glutathione S-transferase (GST)-Ogt fused protein (blue line) in comparison with native GST (green line). Secondary structures: α-helix 3 × 10^−1^, β-sheets 2.2 × 10^−1^ and turn 1.2 × 10^−1^.

The biological role of Ogt under stress conditions was examined by using a functional proteomics approach focused on the identification of its protein partners in vivo. The Ogt protein was first produced by recombinant DNA methodologies in *E. coli* cells as glutathione S-transferase (GST)-fused protein. The recombinant protein was purified by a two-step procedure consisting of affinity chromatography on a gluthathione-conjugated Sepharose column followed by anionic exchange using a MonoQ column (GE Healthcare). The purity of the protein was assessed by SDS-PAGE and its primary structure validated by MALDI mapping. Ogt was digested with trypsin and the resulting peptide mixture was directly analyzed by MALDI-MS/MS tandem mass spectrometry. The mass signals recorded in the spectra were assigned to the anticipated GST-Ogt sequence on the basis of their molecular mass and the fragmentation spectra (Supplementary Materials, Table S1). Circular dichroism analyses were also carried out to verify the correct folding of the protein. Figure 2 shows the corresponding CD spectrum (blue line) in comparison with native GST (green line), displaying 30% α-helix, 22% β-sheets and 12% turn in agreement with the literature data [15] and indicating that Ogt is correctly folded.

Purified recombinant GST-Ogt was then immobilized onto glutathione-conjugated agarose beads to be used as a bait in the functional proteomic experiment. A total *M. smegmatis* cellular extract was then prepared under stress conditions (treatment with 0.03% methyl methane sulfonate (MMS)), and pre-cleaned gluthathione-derivatized beads to remove false positive interactors, i.e., proteins with high affinity for the matrix. The unbound proteins were then incubated with the GST-Ogt derivatized agarose beads and the retained interactors were eluted with an excess of reduced GSH. Proteins retained by the beads during the pre-cleaning step were also eluted and used as control.

Proteins eluted from the sample and the control were fractionated by SDS-PAGE and the gel was stained by colloidal blue coomassie. Protein bands from both the control and the sample lanes were excised from the gel, digested in situ with trypsin and the resulting peptide mixtures were directly analyzed by nanoLC-MS/MS. Mass spectral data were used to search a nonredundant protein database using an in-house version of the Mascot software. Proteins occurring in both the sample and in the control were discarded and only those solely present in the sample lane were considered as putative Ogt interactors.

Among the putative Ogt interactors, a number of proteins involved in stress defense and DNA repair mechanisms were identified, including Ada-AlkA. The occurrence of Ada-AlkA within the Ogt putative interactors led to the hypothesis that these two proteins might form a functional complex to fulfill the biological roles exerted by Ada in the homologous *E. coli* system. These considerations prompted us to further investigate the possible interaction between Ogt and Ada-AlkA.

### 2.2. Expression, Purification and Analysis of Ada-AlkA

Since the Ogt protein was already available in a purified form, a recombinant correctly folded form of Ada-AlkA was needed to test the formation of the putative complex. Recombinant Ada-AlkA was produced in *E. coli* as GST-fused protein following the same procedure as reported above. The recombinant protein was purified by affinity chromatography, anionic exchange and gel filtration and analyzed by SDS-PAGE. Structural analyses were carried out by MALDI mapping (Supplementary Materials, Table S2) and circular dichroism in comparison with native GST and confirmed that recombinant Ada-AlkA had the expected primary structure and was correctly folded. 

**Figure 3:**
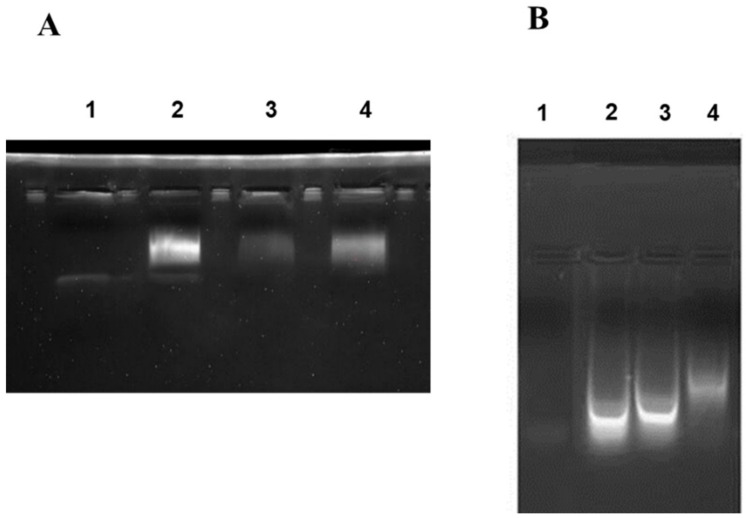
Electrophoresis mobility shift assay (EMSA) performed on Ada-AlkA (**A**) and Ogt (**B**) proteins. A biotin-labeled DNA probe was incubated with each individual protein for 20 min at 25 °C. Panel A: Lane 1, DNA probe. Lane 2, 3 and 4 DNA-probe incubated with different amounts of Ada-AlkA (40, 20 and 60 μM, respectively). Panel B: Lane 1 DNA probe. Lanes 2, 3 and 4 DNA-probe incubated with different amounts of Ogt (10, 50 and 80 μM, respectively).

According to the *E. coli* homologous DNA repair system, Ada-AlkA should be a glycosyl hydrolase with the ability to bind DNA. Therefore, we investigated the DNA binding properties of the recombinant protein by using electrophoresis mobility shift assay (EMSA). The biotin-labeled DNA probe, UP35 was incubated with different amounts of the Ada-AlkA protein for 20 min at 25 °C and the protein-DNA complex was separated on 5% native polyacrylamide gel. Figure 3A shows the resulting gel visualized by UV radiation and clearly displaying an Ada-AlkA-dependent shift in the electrophoresis mobility of the DNA-protein complex compared to the isolated probe.

In Lane 2, a low amount of protein was used for the complex, thus leaving a portion of the probe still free, whereas the oligonucleotide probe was completely involved in the complex at higher concentrations of Ada-AlkA.

These data demonstrated that recombinant Ada-AlkA binds DNA with non-sequence specificity. The same EMSA analysis was carried out with recombinant Ogt to confirm previous data on its DNA binding ability using the random probe (Supplementary Materials, Table S3). [15]. The results shown in Figure 3B demonstrated an Ogt dose-dependent shift of the complex compared to the isolated probe.

### 2.3. Ada-AlkA/Ogt Interaction

Since both Ada-AlkA and Ogt retained their correct tertiary structure and their DNA binding capabilities, we pursued a detailed investigation on the possible interaction between the two proteins both in silico and in vitro. A molecular model of the protein–protein interaction was developed by docking calculations and the results were experimentally confirmed by gel filtration chromatography using the recombinant proteins. 

Ada-AlkA and Ogt models were obtained through the SwissModel webserver, using the *E. coli* AlkA protein (PDB code 3D4V) as a template for the Ada-AlkA model (Supplementary Materials, Figure S1a) and the MTB H37Rv Ogt protein (PDB code 4WXD) as a template for the Ogt model (Supplementary Materials, Figure S1b).

**Figure 4:**
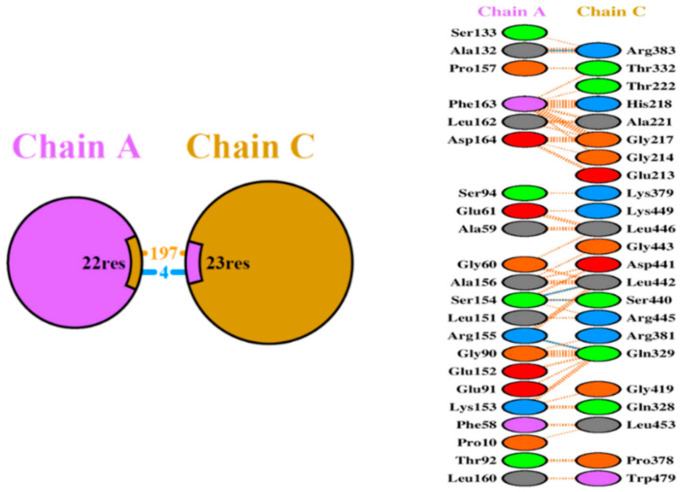
Schematic representation of the identified interactions occurring in the Ada-AlkA/Ogt model. Chain A represents Ogt while Chain C is Ada-AlkA.

The putative structural basis of the protein–protein model Ada-AlkA (Chain C)-Ogt (Chain A) were obtained using the PatchDock Server and the structures were refined through the FireDock Server. Calculations revealed that a stable complex might form with a predicted ΔG = −11.5 Kcal/mol and a dissociation constant, Kd = 3.8 × 10^−9^ M (25 °C). A detailed analysis of the interactions at the protein–protein interface suggested the involvement of 197 non-bonded interactions and four hydrogen bonds: Ala132 Chain A with Arg383 Chain C, Ser154 Chain A with Ser440 Chain C, Ser154 Chain A with Leu442 Chain C and Arg155 Chain A with Gln329 Chain C, as shown in Figure 4.

**Figure 5:**
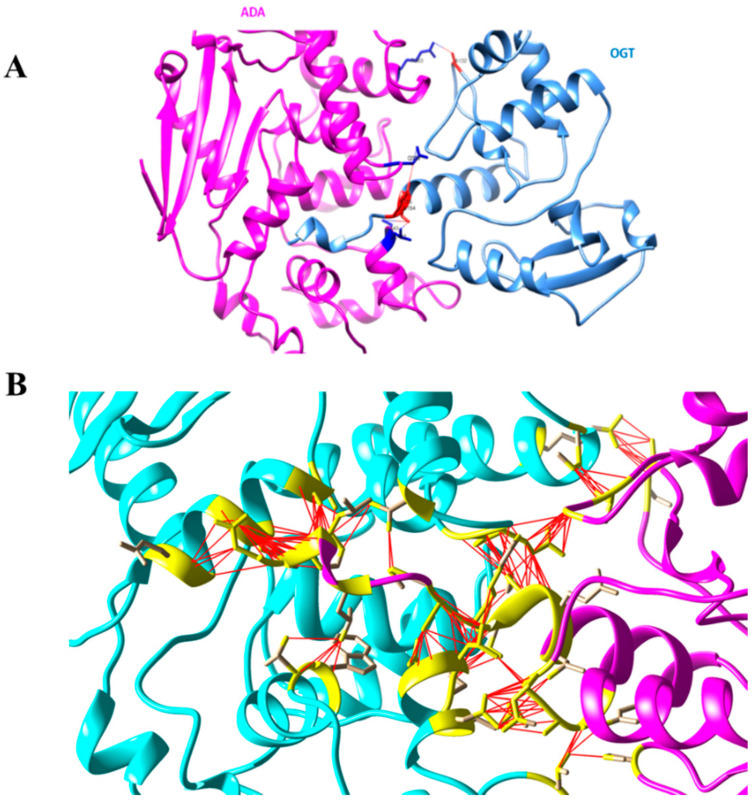
(**A**). Ribbon model of the Ada-AlkA/Ogt complex showing the predicted hydrogen bonds at the protein–protein interface. (**B**). Detailed description of the Ada-AlkA/Ogt interactions. Residues involved in the interaction are in yellow while the red lines represent the connection between residues. Images were generated by the CHIMERA software.

Figure 5 shows an overview of the Ada-AlkA/Ogt complex model (A) and a detailed description of the interactions occurring at the contact surfaces (B).

**Figure 6:**
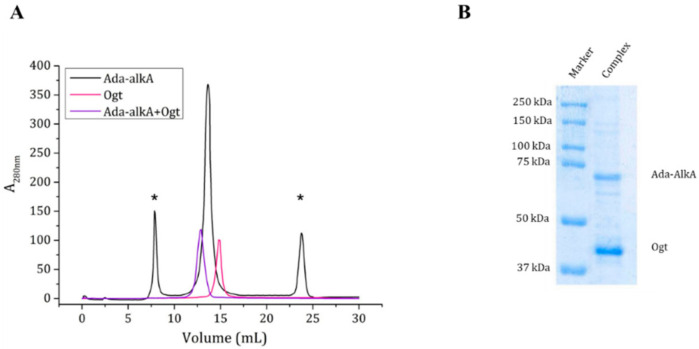
(**A**). Gel filtration chromatography of individual Ada-AlkA (in black) and Ogt (in pink) proteins. Peaks marked with an asterisk represent impurities in the Ada-AlkA preparation. The Ada-AlkA chromatographic peak was recovered, incubated with Ogt and the mixture re-chromatographed (in purple). The chromatogram shows the coelution of Ada-AlkA and Ogt in a single peak. (**B**). The Ada-AlkA/Ogt chromatographic peak was collected and analyzed on 12.5% SDS-PAGE showing two main protein bands with the expected electrophoretic mobility for Ada-AlkA and Ogt. Identification was confirmed by mass spectrometry.

As the docking calculations suggested a possible interaction between Ada-AlkA and Ogt, we confirmed the complex formation on an experimental basis using the recombinant forms of the two proteins in gel filtration assays. A Superdex^®^ 200 column was first calibrated with a mixture of standard proteins and the resulting calibration curve showed a R^2^ value of 0.995 and was then used for further analyses. Purified Ogt and Ada-AlkA proteins were individually subjected to gel filtration chromatography to evaluate their individual exclusion volume and to assess their quaternary structure in solution. The Ogt protein was eluted at a volume of 14.8 mL, while Ada-AlkA showed an elution volume of 13.6 mL. Data processing from the calibration curve returned an apparent molecular weight of about 43 kDa and 79 kDa for Ogt and Ada-AlkA respectively, confirming that both proteins have monomeric structure in solution (Figure 6).

Since, the Ada- AlkA preparation showed the presence of some impurities, the Ada- AlkA peak recovered from the gel filtration column (13.6 mL) was incubated with OGT and the putative complex was analyzed by gel filtration chromatography. Ada-AlkA and Ogt coeluted in a single peak with an elution volume of 12.9 mL. This value was processed with the calibration curve resulting in an apparent molecular mass of 120 kDa, thus confirming the formation of the complex. Figure 6A shows the superimposed profiles of the two individual proteins and the complex. In order to demonstrate the presence of both proteins within the complex, the Ada-AlkA/Ogt chromatographic peak was collected and analyzed by 12.5% SDS-PAGE gel in reducing conditions, which showed the occurrence of two protein bands with the expected electrophoretic mobility for Ada-AlkA and Ogt (Figure 6B). The two bands were excised from the gel digested with trypsin and the resulting peptide mixtures directly were analyzed by LC-MS/MS. 

Mass spectral data were used to search a protein database using the Mascot software, leading to the identification of Ada-AlkA, Swiss Prot code A0R1Z2, and Ogt, Swiss Prot code A0R0A4.

The chromatographic results confirmed the previous docking calculations and proteomics data, which strongly suggested the association of Ada-AlkA and Ogt in a stable complex. Both Ogt and Ada-AlkA are involved in the adaptive response to alkylation damage in DNA caused by alkylating agents. Ada-AlkA catalyzes the hydrolysis of the deoxyribose N-glycosidic bond to excise a number of different methylated DNA bases. Ogt repairs methylated guanines in DNA by transferring the methyl group to a cysteine residue in the enzyme active site, thus fulfilling the same activity exerted by Ada in the homologous *E. coli* system. However, besides its methyltransferase activity, *E. coli* Ada is a sensor protein that upon methylation is activated as a transcriptional regulator that activates the transcription of its own gene and other alkylation resistance genes, while methylation of Ogt seems to inactivate the enzyme. Therefore, the stable association of Ogt with Ada-AlkA suggests that the complex might exert both methyltransferase and N-glycosyl hydrolase enzymatic activities and have a transcriptional activator role upon methylation.

### 2.4. Expression, Purification and Structural Analyses of FadE8

Finally, we investigated FadE8, a *M. smegmatis* protein belonging to the adaptative response mechanism homologous to the *E. coli* AidB protein. Recombinant FadE8 was expressed in *E. coli* bearing a N-terminal His tag and purified to homogeneity by affinity chromatography on a Ni-derivatized column. The primary structure of the recombinant protein was verified by MALDI mapping strategy (Supplementary Materials, Table S4) and its correct folding assessed by circular dichroism analyses.

*E. coli* AidB is a flavoprotein belonging to the acyl-Coenzyme A dehydrogenase family, which has shown weak dehydrogenase activity and unspecific DNA binding. A detailed characterization of recombinant FadE8 was then carried out to explore the interaction with flavin adenine dinucleotide (FAD) using docking calculations, the enzymatic activity and the DNA binding capability. 

**Figure 7:**
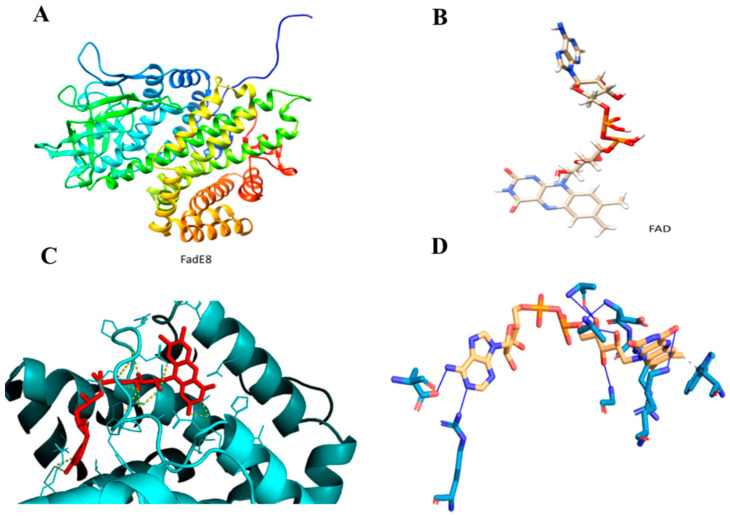
(**A**). FadE8 model obtained with I-TASSER Server. (**B**). Flavin adenine dinucleotide (FAD) model obtained with the LigParGen Server. (**C**). Zoom image of the FadE8/FAD model. The protein is shown in cyan while the ligand is shown in red. (**D**). Predicted interactions between FadE8 and the FAD ligand using the Protein-Ligand Interaction Profiler (PLIP) Server.

Molecular docking analysis was performed by modeling FadE8 with the I-TASSER Server to obtain the best model with a C-score value of 1.83, an estimated TM-score of 0.97 ± 0.05 and an estimated RMSD score value of 3.7 ± 2.5 Å (Figure 7A). Figure 7B shows the FAD ligand model obtained with the LigParGen Server. The Acyl-CoA dehydrogenase from *Brucella melitensis* in complex with FAD (PDB code 5EZ3 A) was used as a template to model the FadE8-FAD complex. The FadE8-FAD model was obtained using the PatchDock Server and the structures were refined through the FireDock Server. Calculations revealed the occurrence of a stable protein-ligand complex with a Gibbs free energy of ΔG = −7.4 Kcal/mol was predicted using the PRODIGY webserver (Figure 7C). A detailed analysis of the interactions at the protein/ligand interface suggested the involvement of both hydrophobic interactions and hydrogen bonds. In particular, Trp36 and Arg107 are involved in hydrophobic interactions with the ligand while Ala101, Ala104, Asp106, Gly109, Lys117, Glu288 and Arg371 residues establish hydrogen bonds with the FAD molecule (Figure 7D).

As the docking calculations confirmed the interaction of FadE8 with FAD, we tested the dehydrogenase activity of the recombinant protein. FadE8 was incubated in the presence of FAD and isovaleryl-CoA as substrate using 2, 6 dichlorophenolindophenol (DCPIP) as the final electron acceptor. The enzymatic activity was monitored by the change in absorbance at 600 nm over time. Table 1 reports the results obtained compared with human isovaleryl-CoA dehydrogenase and the AidB activity reported in the literature [17].

As expected, FadE8 displayed weak dehydrogenase activity very similar to AidB and much less than the human enzyme, suggesting that the specific FadE8 substrate might be different from Acyl-CoA molecules as already observed for AidB [11].

**Figure 8:**
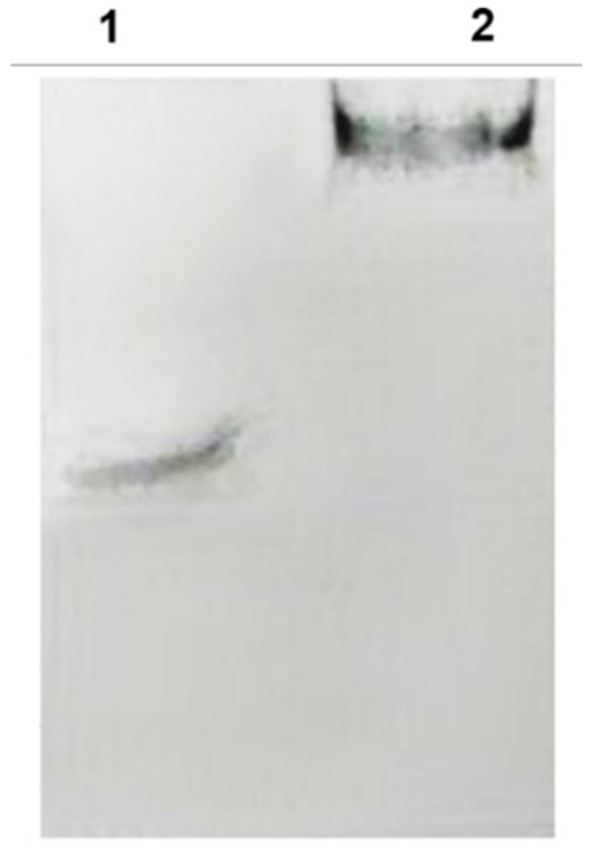
EMSA assay performed on FadE8 protein. A biotin-labeled DNA probe was incubated with the protein for 20 min at 25 °C. Lane 1, DNA probe. Lane 2 DNA-probe incubated with 40 µM FadE8 showing the retardation shift.

Finally, the DNA binding properties of recombinant FadE8 were investigated by EMSA. The biotinylated UP DNA fragment was incubated with the protein for 20 min at 25 °C and the protein-DNA complex was separated on 5% native polyacrylamide gel. A clear retardation shift in the electrophoretic mobility of the DNA-protein complex was observed compared to the free probe (Figure 8).

These data confirmed that similarly to its homologous AidB, recombinant FadE8 retained its capability to bind DNA with non-sequence specificity.

## 3. Materials and Methods

### 3.1. Recombinant Production of FadE8, Ogt and Ada-AlkA Proteins

The *fadE8*, *ogt* and *ada-alkA* genes were amplified from *M. smegmatis* genome by polymerase chain reaction (PCR). The *fadE8* gene was cloned into the pET22b-c-myc vector containing the coding sequence for the corresponding recombinant protein fused to a 6-histidine tag at the C-terminus. The *ada-alkA* and *ogt* genes were cloned in pGEX-4T1 vector containing sequences encoding for the corresponding recombinant proteins fused to GST at the N-terminus. Plasmid construction was verified by automated DNA sequencing.

Expression of the recombinant proteins was carried out in BL21 *E. coli* cells, in LB medium at 37 °C with 100 μg/mL kanamicin for FadE8 and 100 μg/mL ampicillin for Ogt and Ada-AlkA in order to select the strain of interest. Expression of recombinant proteins was induced in the exponential phase with isopropyl-thio-β-D-galactoside (IPTG) at a final concentration of 1 mM for FadE8 and 0.1 mM IPTG for Ogt and Ada-AlkA. Cultures were grown at 20 °C for 16 h and cells were then retrieved by centrifugation at 5000× *g* rpm for 20 min at 4 °C.

The cellular pellets were resuspended into two different buffers, 0.1 M Na_2_HPO_4_, 0.15 M NaCl, 1 mM PMSF, pH 7.4 for Ogt and Ada-AlkA and 20 mM Na_2_HPO_4_, 0.5 M NaCl, 20 mM imidazole, 1 mM PMSF, pH 7.4 for FadE8.

Cells were lysed by sonication for 20 min and the sample was centrifuged at 13,000× *g* rpm for 30 min at 4 °C, allowing the separation of the insoluble fraction, which contained inclusion bodies, from the soluble sample. Proteins were purified from the soluble fraction. 

His_6_-FadE8 was purified by affinity chromatography on His-Select Nickel Affinity beads (Thermo Fisher, Waltham, Massachusetts, USA) and eluted with 500 mM imidazole in 20 mM Na_2_HPO_4_, 0.5 M NaCl, pH 7.4.

GST-Ogt was purified through two purification steps including affinity chromatography on glutathione agarose beads (Sigma-Aldrich, Darmstadt, Germany) eluted with 1 mM reduced glutathione in 50 mM Tris-HCl pH 9.0, followed by anionic exchange chromatography on a HiTrap Q-HP, 5 mL column (GE Healthcare, Chicago, IL, USA), connected to a fast liquid protein chromatography system. The protein was eluted with 1 M NaCl in 50 mM Tris-HCl pH 8.

Purification of GST-Ada-AlkA was performed through a three-step procedure consisting of affinity chromatography on glutathione agarose beads (Sigma-Aldrich, Darmstadt, Germany ) eluted with 10 mM reduced glutathione in 50 mM Tris-HCl pH 9.0, anionic exchange chromatography on HiTrap Q-HP, 5 mL column (GE Healthcare) eluted with 1 M NaCl in 50 mM Tris-HCl pH 8.0 and gel filtration on a Superdex^®^ 200 10/300 GL column connected to a fast liquid protein chromatography system, eluted with 50 mM Tris-HCl pH 8.0, containing 150 mM NaCl.

Protein concentration was determined with the Bradford Reagent from Sigma, using BSA as a standard [18]. Protein purity was verified by SDS-PAGE and their primary structure was validated by MALDI mapping strategy on a 5800 MALDI-TOF/TOF instrument (ABI Sciex, CA, USA) Circular dichroism analyses was performed to verify the correct folding of the proteins using a JASCO J-715 spectropolarimeter equipped with a Peltier thermostatic cell holder (Model PTC-348WI) and a 1 cm optical path-length quartz cell. CD spectra were acquired in the range 190–250 nm, with three accumulations performed for each measurement, at a scanning speed of 50 nm/min and data pitch of 1.0 nm.

The quaternary structure of Ada-alkA and Ogt was assessed by gel filtration chromatography on Superdex^®^ 200 10/300 GL eluted with 50 mM Tris-HCl pH 8.0 containing 150 mM NaCl.

### 3.2. Protein-DNA Interaction

The interaction of Ada-alkA, Ogt and FadE8 with DNA was investigated by EMSA experiments using biotin-labeled DNA probes, fragments UP35, UP(Primm) and a random probe (the MALDI-MS spectrum is reported in Supplementary Materials, Figure S2, a kind gift from Dott.ssa Musumeci). Oligonucleotide sequence probes are shown in Supplementary Materials, Table S3).

Sense and antisense oligonucleotides were annealed by incubation at 95 °C for 5 min and then gradual cooling to room temperature. Proteins were incubated with the probes for 20 min at 25 °C in 20 µL of 25 mM HEPES pH 7.6, containing 50 mM KCl, 12.5 mM MgCl_2_, 1 mM DTT, 20% glycerol, and 0.1% triton. Protein–DNA complexes were separated on 5% native polyacrylamide gel (29:1 cross-linking ratio) in 45 mM Tris pH 8.0, containing 45 mM boric acid, 1 mM EDTA at 200 V (20 V/cm) at room temperature. Visualization of DNA samples was carried out using UV radiation.

### 3.3. Isovaleryl-CoA Dehydrogenase Activity of FadE8

The dehydrogenase activity of FadE8 was evaluated by incubation with 2 mM isovaleryl-CoA (Sigma-Aldrich, Darmstadt, Germany) as substrate and 0.1 mM 2, 6 dichlorophenolindophenol (DCPIP) as the terminal electron acceptor in a final volume of 300 μL at room temperature in 200 mM phosphate buffer, pH 8.0. Enzymatic activity was determined by monitoring the change in absorbance at 600 nm using a Beckman DU 7500 spectrophotometer assuming a molar extinction coefficient of 20.6 mM^−1^ cm^−1^ for DCPIP (13).

### 3.4. FadE8-Flavin Adenine Dinucleotide Molecular Docking Analysis

The ability of FadE8 to bind the flavin adenine dinucleotide (FAD) coenzyme was evaluated by docking analyses. The FadE8 protein was modeled using the I-TASSER Server [19,20,21]. Each model is associated with a C-score whose value ranges from −5 to +2. The higher this value the better the model. TM-score and RMSD are known standards for measuring structural similarity between two structures. A TM-score value > 0.5 indicates a model of correct topology while a score < 0.17 means a random similarity [22]. FAD model was obtained using the LigParGen Server [23,24,25] exploiting the Isomeric SMILES Code, Cc1cc2nc3c(nc( =O)[nH]c3=O)n(C[C@H](O)[C@H](O)[C@H](O)CO[P@](O)(=O)O[P@@](O)(=O)OC[C@H]3O[C@H]([C@H](O)[C@@H]3O)n3cnc4c(N)ncnc34)c2cc1C, from the RCSB PDB (Protein DataBase). The protein-ligand model was constructed using the PatchDock Server [26] and the structures were then refined with the FireDock Server [27], which also provided the global energy, the attractive and repulsive Van der Waals (VdW) forces and the atomic contact energy (ACE) values of the complex. The Protein-Ligand Interaction Profiler (PLIP) Server [28] was used to define the interactions between the FadE8 protein and the ligand. Finally, the ΔGnoelec binding affinity of the complex was predicted using the PRODIGY webserver using the “No Electrostatics Prediction” protocol [29]. All the figures were generated through UCSF CHIMERA software [30] and the PyMOL Molecular Graphics System, Version 1.2r3pre, Schrödinger, LLC.

### 3.5. Isolation of Ogt Complexes in Mycobacterium smegmatis

The GST-Ogt fused protein was produced in *E. coli*, and purified according to the procedure described above. The purified recombinant protein was immobilized on glutathione-derivatized agarose beads.

A total *M. smegmatis* cellular extract was prepared by growing mycobacterial cells in LB medium containing 100 μg/mL ampicillin and 0.05% tween 80. At a value of 0.4 OD/mL the culture was treated with sub-inhibitory concentrations (0.03%) of the alkylating agent methyl methane sulfonate (MMS). After 3 h, cells were recovered by centrifugation at 4000× *g* rpm at 4 °C for 15 min and the pellets were solubilized in 50 mM Na_2_HPO4 pH 7.4 containing 150 mM NaCland1 mM PMSF. Cells were disrupted by French press and centrifuged at 14,000× *g* rpm for 30 min at 4 °C. *M. smegmatis* protein extract was pre-cleaned by incubation with reduced glutathione-agarose beads to remove non-specific binding proteins. The unbound proteins were incubated with the GST-Ogt derivatized beads for 2 h at 25 °C. After extensive washing with 50 mM Na_2_HPO_4_ containing 150 mM NaCl and 1% Triton, the proteins specifically bound to the GST-Ogt bait were eluted with 20 mM Tris-HCl buffer pH 8.0 containing 1 mM reduced glutathione-proteins from the pre-cleaning step were also eluted and used as control.

Proteins from the sample and the control were fractionated by 10% SDS-PAGE and the gel bands were digested in situ with trypsin. The resulting peptide mixtures were directly analyzed by liquid chromatography/tandem mass spectrometry (LC-MS/MS) on a LTQ Orbitrap XL system (Thermo Fisher, Waltham, Massachusetts, USA) equipped with a nano-LC. The elution was accomplished with 95% acetonitrile, 5% water and 0.1% formic acid as eluent. Mass spectral data were used for database search by an in-house version of the Mascot software leading to the identification of the proteins. Proteins that occurred in both the sample and the control were discarded and those found solely in the sample were considered as putative Ogt interactors.

### 3.6. Ada-AlkA and Ogt Molecular Docking Analysis

The putative interaction between Ogt and on Ada-AlkA was determined by molecular docking calculations. Both proteins were modeled using the SWISS MODEL Server [31] and the model of the Ada-AlkA-Ogt complex was obtained using the PatchDock Server [26]. The structure was refined with the FireDock Server [27], which also provided the global energy, the attractive and repulsive Van der Waals (VdW) forces and the atomic contact energy (ACE) values of the complex. The amino acids occurring at the protein interface and the molecular interactions were identified by the PDBsum Server [32,33,34]. The Gibbs free energy, ΔG, and the dissociation constant, Kd, of the complex were predicted using the PRODIGY webserver [35,36]. All the figures were generated by the UCSF CHIMERA software [30].

### 3.7. Validation of Ada-AlkA and Ogt Complex

The formation of the Ada-AlkA/Ogt complex was evaluated by gel filtration chromatography. The two purified proteins were incubated in 1:1 ratio in 50 mM Tris-HCl pH 8.0 containing 20 mM NaCl for 2 h at 25 °C and the complex was chromatographed on a Superdex^®^ 200 10/300 GL column. Calibration of the column was performed using thyroglobulin (669 kDa), apoferritin (443 kDa), β-amylase (200 kDa), albumin (66 kDa) and carbonic anhydrase (29 kDa) as molecular mass markers. The collected fractions were analyzed by SDS-PAGE and the bands corresponding to Ada-AlkA and Ogt proteins were digested in situ with trypsin and identified by LC-MS/MS using an HPLC-Chip/Q-TOF 6520 (Agilent Technologies, Santa Clara, CA, USA) to confirm the identity of the two proteins.

## 4. Conclusions

This work focused on investigating the specific role of proteins involved in the DNA repair mechanism in *M. smegmatis*, a non-pathogenic mycobacterium. Although this mechanism has been widely described in *E. coli*, it is still pretty unknown in mycobacteria, which show a different genetic organization. With the aim of describing the DNA repair mechanism in mycobacteria, we first investigated the protein components of the *M. smegmatis* system. Ogt, Ada-AlkA and FadE8 proteins were cloned and expressed in *E. coli* and validated for their amino acid sequence and the correct folding.

The functional proteomics experiment was aimed at identifying the Ogt protein partners and the results suggested that this protein specifically interacts with Ada-AlkA. Formation of the Ogt/Ada-AlkA complex was then confirmed by docking calculations and gel filtration experiments. Since Ogt is homologous to the B domain of *E. coli* Ada protein, its specific association with Ada-AlkA might restore the same functional activity shown by the Ada protein in *E. coli*, even though they are part of different genes. Therefore, the presence of Ogt and Ada-AlkA in the complex might provide the complex with multiple biological functions that enable it to bind DNA and to exert both methyltransferase and N-glycosidase enzymatic activities. Moreover, it can be expected that the Ada-AlkA moiety is converted to a sensor protein that acts as a positive transcriptional regulator for the other genes involved in the adaptive response to DNA alkylation damage in *M. smegmatis*. However, further genetic investigations are needed to confirm this hypothesis.

Finally, we were able to outline a structural and functional analysis of FadE8 protein and to determine that this protein and its *E. coli* homologous AidB are not only structurally but also functionally related. We demonstrated that FadE8 is able to bind the FAD coenzyme, thus showing a dehydrogenase activity similar to AidB and it is endowed with a specific DNA binding capability.

The investigation of the DNA repair system in *M. smegmatis* allowed us to characterize proteins that are also present in MTB and play key roles in survival and protection mechanisms. Since these proteins are absent in humans, they might represent excellent targets for possible new therapeutic approaches.

## Figures and Tables

**Table 1:** Enzymatic activity of recombinant FadE8 in comparison with *E. coli* AidB and human Acyl-CoA dehydrogenase.

Protein	Isovaleryl-CoA Dehydrogenase Activity (μmol min^−1^/mg Protein)
AidB *(E. coli)*	0.12 ± 0.01
FadE8 *(M. smegmatis)*	0.30 ± 0.01
Human Acyl-CoA dehydrogenase	8.2 to 11.7

## Supplementary Materials

The following are available online at https://www.mdpi.com/1422-0067/21/15/5391/s1, Figure S1: Ada and Ogt protein models; Figure S2: MALDI-MS spectrum. MALDI-MS spectrum of the Random oligonucleotide probe in the linear mode showing the expected mass value (7632.21 Da). Table S1: Peptide mapping of the GST-Ogt fused protein by MALDI-TOF; Table S2: Peptide mapping of the GST- Ada-AlkA fused protein by MALDI-TOF; Table S3: Oligonucleotide probes used in the EMSA experiments; Table S4: Peptide mapping of the FadE8 protein by MALDI-TOF.

## Abbreviations

O^6-^MeG	O^6^-methylguanine
N^7-^MeG	N^7^-methylguanine
N^3^-MeA	N^3^-methyladenine
N^1^-MeA	N^1^-methyladenine
N^3^-MeC	N^3^-methylcytosine
O^4-^MeT	O^4^-methyltimine
DCPIP	2,6 dichlorophenolindophenol
MTB	Mycobacterium tuberculosis
GST	Glutathione S-transferase
GSH	Glutathione
EMSA	Electrophoretic mobility shift assay
CD	Circular dichroism
PDB	Protein Data Bank
FAD	Flavin adenine dinucleotide
PCR	Polymerase chain reaction
IPTG	Isopropyl-thio-β-D-galactoside
LC-MS/MS	Liquid chromatography/tandem mass spectrometry
