# Supplementary figures and images for: Characterization of the Proteins Involved in the DNA Repair Mechanism in M. smegmatis
Source: Int J Mol Sci. 2020 Jul 29;21(15):5391. doi: 10.3390/ijms21155391 (PMC7432924; doi:10.3390/ijms21155391)

a

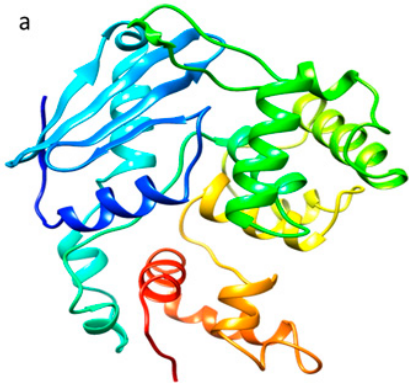

b

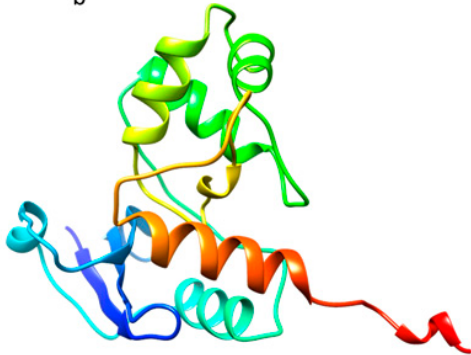

Supplement: Supplementary file 1 [file ijms-21-05391-s001.pdf]
